# Gastrotomy followed by gastrorrhaphy as a reliable and more physiologic technique for inducing peritoneal adhesion in rats

**DOI:** 10.1590/0100-6991e-20233453-en

**Published:** 2023-07-19

**Authors:** ANTONIO AUGUSTO RIBEIRO DIAS PIRES, CHRISTINA MAEDA TAKIYA, PAULO CESAR SILVA, JOSÉ EDUARDO FERREIRA MANSO

**Affiliations:** 1 - Universidade Federal do Rio de Janeiro, Departamento de Cirurgia Experimental - Rio de Janeiro - RJ - Brasil; 2 - Hospital Naval Marcílio Dias - Rio de Janeiro - RJ - Brasil

**Keywords:** Models, Animal, Tissue Adhesions, Inflammation, General Surgery, Modelos Animais, Aderências Teciduais, Inflamação, Cirurgia Geral

## Abstract

**Objective:**

this research objective was to develop a new peritoneal adhesion animal model that would lead to adhesions formation in all operated animals, simple and reproducible, associated with maintenance the animal’s health.

**Methods:**

eighteen adult male Wistar rats (Rattus norvegicus) were randomly distributed into three groups: Control Group (anatomical and clinical parameters), Sham Group (delicate manipulation of the stomach and exposure of the peritoneal cavity to ambient air) and Surgery Group (gastrotomy followed by gastrorrhaphy). The animals were analyzed and classificated macroscopically according to two adhesion classification models and differences between groups were considered significant when p<0.05.

**Results:**

the six animals in the control group had no peritoneal adhesions, three of the six animals in the sham group had focal peritoneal adhesions, and all animals in the surgery group (gastrotomy followed by gastrorraphy) had firm peritoneal adhesions. All adhesions found were macroscopically quantified and microscopically confirmed, without carrying out a microscopic classification of the adhesions.

**Conclusion:**

the new model developed of gastrotomy followed by gastrorrhaphy, proved to be safe and efficient to induce and study peritoneal adhesions.

## RESUMO

**Objetivo:**

o objetivo deste estudo foi criar um novo modelo animal de indução de aderências peritoneais capaz de levar à formação de aderências em todos os animais operados, simples e reprodutível, associado a manutenção da saúde dos animais.

**Métodos:**

Dezoito ratos machos, adultos, da linhagem Wistar (Rattus norvegicus) foram distribuídos aleatoriamente em três grupos: Grupo Controle (parâmetro anatômico e clínico), Grupo Sham (manipulação delicada do estômago e exposição de cavidade peritoneal ao ar ambiente) e Grupo Cirurgia (gastrotomia seguida de gastrorrafia). Os animais foram submetidos à análise e classificação macroscópicas, seguindo dois modelos de classificação de aderências. As diferenças entre os grupos foram consideradas estatisticamente significantes se p<0,05.

**Resultados:**

os seis animais do grupo controle não apresentavam aderências peritoneais, três dos seis animais do grupo sham apresentavam aderências peritoneais focais e todos os seis animais do grupo cirurgia (gastrotomia seguida de gastrorrafia) apresentavam aderências peritoneais firmes. Todas as aderências encontradas foram quantificadas macroscopicamente e confirmadas microscopicamente, sem a realização de classificação microscópica das aderências.

**Conclusão:**

o novo modelo desenvolvido, de gastrotomia seguida de gastrorrafia, mostrou-se seguro e eficiente para induzir e estudar aderências peritoneais.

## INTRODUCTION

Peritoneal adhesions occur when there is formation of scar tissue connecting two or more previously separated peritoneal structures, and may appear between the abdominal organs or between them and the parietal peritoneum. It is a frequent disease that causes numerous postoperative complications, generating enormous costs for the health system, reaching billions of dollars in the United States^1^ ^-^ ^8^. In addition to cost, peritoneal adhesions can cause chronic pain, female infertility, and intestinal obstruction^3^ ^,^ ^4^ ^,^ ^7^ ^-^ ^10^, with greater difficulty in new abdominal surgical approaches^10^, increasing operative time and the risk of injury to intraperitoneal organs^3^, leading to a higher risk of infections^7^. Around 440,000 surgeries are performed annually in the United States to treat peritoneal adhesions^6^, 300,000 of them to treat small bowel obstruction^9^, and around 2,000 deaths per year are related to this pathology in that country^7^.

Numerous studies are published annually on peritoneal adhesions, requiring the use of animal models to induce adhesions when in the context of experimental surgery. The methods created and replicated in previous studies^1^ ^,^ ^2^ ^,^ ^4^ ^,^ ^10^ ^-^ ^15^ may not be able to induce the formation of peritoneal adhesions, even with the correct reproduction of the animal models described, and it is rare to find a publication in which the animal model used reached the formation of adhesions in all animals. In addition, most animal models proposed to induce peritoneal adhesions rely on performing excessive damage, therefore contradictory to what is done in surgical procedures, which seek to minimize unnecessary damage.

The objective of this study was to create a new animal model of induction of peritoneal adhesions, capable of leading to the formation of peritoneal adhesions in all operated animals, being simple and reproducible, and maintaining the animals’ health.

## METHODS

We used 18 adult, male, Wistar rats (Rattus norvegicus), with a body weight of 300g (±80g), randomly allocated into groups, originating from, and maintained in, the bioterium of the Experimental Surgery Laboratory of the Federal University of Rio de Janeiro, in a controlled environment, with constant temperature (22°C ± 2°C), light/dark cycles of twelve hours, receiving water and balanced chow ad libitum. Monitoring of animal welfare and calculation of the sample size were performed according to CONCEA Normative Resolution No. 2516. Preoperative fasting was not used and the same surgeon operated all animals. The project was approved by the Ethics Committee for the Use of Animals in Research.

The animals underwent intraperitoneal general anesthesia with ketamine (100mg/kg) and xylazine (10mg/kg) through a puncture in the lower right quadrant of the abdomen, using a 13 x 0.45mm needle. The puncture was performed with the animals in a head down position to minimize the possibility of inadvertent injury to the intestinal loops. After anesthesia, trichotomy and antisepsis of the abdominal wall were performed with povidone iodine, so that the surgical procedures could be started. The operations took place at the Experimental Surgery Laboratory of the Universidade Federal do Rio de Janeiro.

The animals were randomly distributed into three groups, being kept throughout the postoperative period in the same environment:

Control group: six animals submitted to no procedure until euthanasia and necropsy. This group was used as an anatomical and clinical parameter of a normal animal.

Sham Group: six animals underwent a median laparotomy of 5.0cm, with manipulation of the liver, stomach, and omentum, keeping the peritoneal cavity open for twenty minutes, the average time of operations in the surgery group. The abdominal wall was then closed with continuous, 5.0 polypropylene suture.

**Figure 1:**
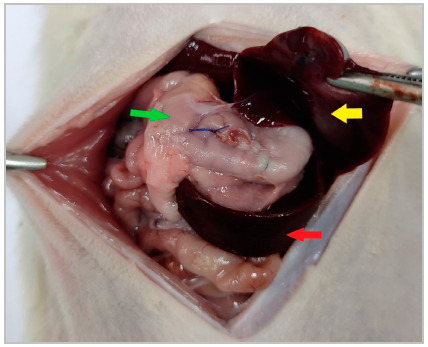
Surgery group rat, submitted to the gastrostomy followed by gastrorraphy model. Yellow arrow liver, green arrow stomach, red arrow spleen. It can be noticed, in the center of the suture line, area with exposed gastric mucosa.

Surgery group (gastrotomy followed by gastrorrhaphy): six animals underwent a 5.0cm median laparotomy. The stomach was exteriorized and a 1.0cm longitudinal incision was made in the anterior wall of the antrum/gastric body, followed by immediate closure with continuous, 5.0 polypropylene suture. The use of seven semi-knots was adopted as standard both for the beginning and for the end of the continuous suture, which ran from the serosa to the submucosa, sparing the mucosa, the amount of tissue between the edge of the incision and the stitch being between 1 and 2mm, enough to minimize the risk of dehiscence (Figure 1). The unintentional exteriorization of part of the gastric mucosal segments through the suture line was allowed, occurred in all animals, and affected less than 10% of the suture length. During the gastrotomy, when gastric contents leaked, the secretion was immediately removed with sterile gauze, preventing dissemination to the peritoneal cavity. The abdominal wall was closed with continuous, 5.0 polypropylene suture.

Postoperative care - The diet was restarted twelve hours after surgery, with water and food ad libitum. Postoperative analgesia was performed with ibuprofen diluted in the offered water, at a concentration of 0.2mg per milliliter of water. All animals were observed for signs of pain and stress and had their well-being assessed by the vivarium caretaker, in accordance with the CONCEA Normative Resolution No. 2516.

Euthanasia - Performed on the 14^th^ postoperative day by pharmacological overdose of ketamine (300mg/kg) and xylazine (30mg/kg), through a puncture in the lower right quadrant of the abdomen, using a 13 x 0.45mm needle, after the animals had been positioned in a head down position. No new trichotomy or antisepsis was performed. After the cardiorespiratory arrest, a U-shaped laparotomy was carried out to avoid any damage to the formed peritoneal adhesion. The abdominal cavities were photographed with a Samsung^®^ S9+ cell phone camera.

Two macroscopic grading scales for peritoneal adhesions, frequently mentioned in the literature, were used to classify adhesions^12^ ^,^ ^13^. These scales were adapted from those published by Evans et al.^13^ (Table 1) and Nair et al.^12^ (Table 2), and allowed the assessment of the quantity, intensity, and quality of adhesions.

**Table 1:** Adapted from Evans et al. ^13^ .

Score	Description of adhesion aspects
0	Absence of adhesions
1	Laminar adhesions that are separated spontaneously
2	Firm adhesions but separated with traction
3	Dense adhesions requiring sharp instruments to separate adherent structures

**Table 2:** Adapted from Nair et al. ^12^ .

Score	Description of adhesion aspects
0	Absence of adhesions
1	Single band of adhesion between viscera or between a single viscera and the abdominal wall
2	Two adhesion bands between viscera or between viscera and abdominal wall
3	More than two bands of adhesion between viscera or between viscera and abdominal wall or block adhesion without involvement of the abdominal wall
4	Direct adherence of the viscera to the abdominal wall, regardless of the number and length of the adhesion bands.

Histology - The adhered structures were resected en bloc to avoid rupture of peritoneal adhesions and were subsequently fixed in a 10% formaldehyde solution, cleaved by the author, inserted in histological cassettes, and processed for histology. The blocks were cut using a rotary microtome and the cuts were fixed on histological slides and stained with Hematoxylin-Eosin (HE) and Picrosirius Red (PR) for collagen visualization. The histological slides were scanned with a Leica^®^ Aperio CS2 slide scanner and analyzed under the E 800 Nikon^®^ microscope. The procedures were performed at the Serviço de Anatomia Patológica do Hospital Naval Marcílio Dias e no Laboratório de Imunopatologia do Instituto de Biofísica Carlos Chagas Filho (Universidade Federal do Rio de Janeiro).

In this study, we used macroscopic evaluation to verify the presence of postoperative peritoneal adhesions and to perform the analysis, classification, and quantification of these adhesions. We applied microscopic evaluation to confirm that the macroscopically found adhesions were formed by scar tissue, composed of collagen, inflammatory cells, and neoformed vessels, and not by bands of non-degraded fibrin, which would not be considered adhesions, despite being a visible structure connecting intraperitoneal organs. There was no microscopic analysis to quantify or grade adhesions.

Statistical analyzes - We analyzed the data with the GraphPadPrism v. 5.0 (GraphPad Software, La Jolla, CA, USA). We performed the Non-parametric, One way-ANOVA (Kurskal-Wallis) test followed by Dunn’s post hoc test. Differences between groups were considered statistically significant when p<0.05.

## RESULTS

**Figure 2:**
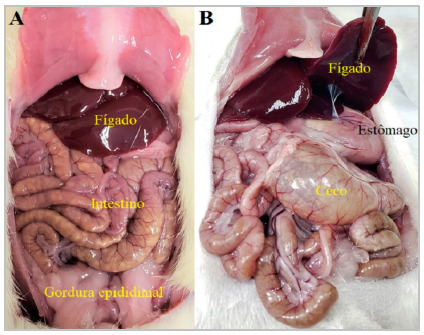
Control group rat. Normal anatomic parameter. No adhesions.

**Figure 3:**
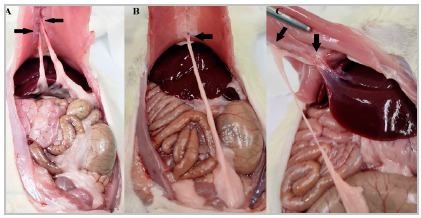
Sham group rat,black arrows point to the adhesions, no other adhesions on the abdominal cavity. A- Between the omentum and the laparotomy scar. B- Between the epididymal fat and the laparotomy scar. C- Between the epididymal fat and the laparotomy scar and between the liver and the laparotomy scar (through a thin band of adherence).

**Figure 4:**
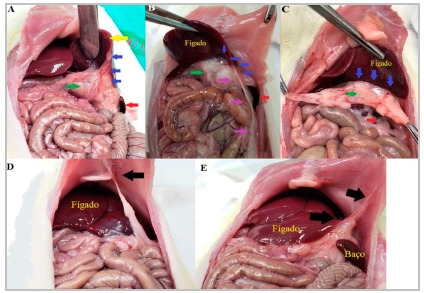
Surgery group rats. A, B and C - Yellow arrow liver, red arrow spleen, green arrow stomach with omentum, blue arrows points to adhesion area between liver, stomach and omentum, purple arrows points to the epididymal fat that extends from the pelvis to the liver-stomach-omentum adhesion.

No peritoneal adhesions was found in any of the animals in the study at the time of the first laparotomy. The six animals in the control group remained clinically well, with no signs of pain or stress, and with satisfactory weight gain. We observed no peritoneal adhesions at their necropsy (Figure 2). The six animals in the sham group remained clinically well, with no signs of pain or stress, and with satisfactory weight gain until the 14^th^ postoperative day, when euthanasia was performed. During necropsy, three animals in this group had peritoneal adhesions (Figure 3). The six animals in the surgery group remained clinically well, with no signs of pain or stress, and with satisfactory weight gain until the 14^th^ postoperative day, when euthanasia was performed. During necropsy, all animals in this group had firm and dense adhesions between the stomach, liver, and omentum (Figure 4). In addition, four rats had extension of the adhesions to the abdominal wall (Figures 4D and 4E).

When evaluating the groups according to the Evans^13^ (Table 1) and Nair^12^ (Table 2) scales, the six animals in the control group did not present adhesions, being attributed zero in both scales.

Three animals from the sham group had adhesions between the omentum and the abdominal wall, or between the epididymal fat and the abdominal wall, and in one of these, there was also an adhesion band between the liver and the abdominal wall (Figure 3). The three animals in the sham group that did not have adhesions were assigned zero on both scales. The three animals in this group that had adhesions were assigned grade 2 according to the Evans scale, as they had adhesions that could be undone by traction, but when evaluated according to the Nair scale, two animals had grade 2 adhesions due to the presence of two adhesion area (Figures 3A and 3C), and one animal had grade 1 because it had only one adhesion area (Figure 3B).

In the surgery group, when assessed using the Evans scale, Table 3 was assigned to all animals, as adhesions could only be separated using sharp instruments. When evaluated using the scale proposed by Nair, three of the six animals had Table 4 adhesions, with the viscera (liver) adhering directly to the abdominal wall, and the remaining three animals had firm adhesions between the stomach, liver, and omentum, creating an inseparable block of adhesions between the structures and being assigned grade 3 (Figure 4).

**Table 3:** Results according to the Evans score.

	Group control	Sham group	Surgery Group
Rat 1	0	0	3
Rat 2	0	0	3
Rat 3	0	2	3
Rat 4	0	2	3
Rat 5	0	2	3
Rat 6	0	0	3

**Table 4:** Results according to Nair score.

	Group control	Sham group	Surgery Group
Rat 1	0	0	3
Rat 2	0	0	4
Rat 3	0	2	3
Rat 4	0	1	4
Rat 5	0	2	3
Rat 6	0	0	4

**Figure 5:**
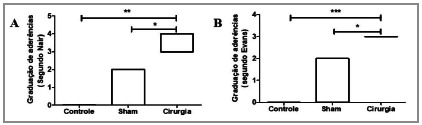
Statistical analysis between groups. Result comparison between sham and control groups without statistical significance. Comparison between the surgery group and the control group and between the surgery group and the sham group showed statistical significance. A- Using Nair’s graduation. *p<0.001. *p<0.05. B- Using Evans’s graduation. **p<0.0001. *p<0.05.

When statistically analyzing the results of each group, compiled in tables 3 and 4, the comparison between the control group and the sham group did not achieve statistical significance in any of the evaluated macroscopic scales, but when comparing the control group with the surgery one, there was statistical significance both on the Nair (p<0.001) and on the Evans (p<0.0001) scales. Smilarly, we obtainded statistical significance when comparing the sham group with the surgery one (p<0.05). The graphical result of the statistical analysis can be seen in Figures 5A and 5B, representing the Nair and Evans scores, respectively. Based on these results, the presented model was effective in inducing extensive and stable peritoneal adhesions, which were present in all animals.

**Figure 6:**
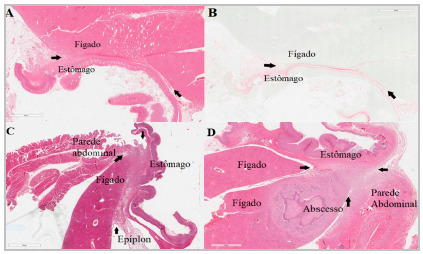
Surgery group rats. A- Slide stained with Hematoxylin and Eosin showing adhesion between the liver and the stomach, pointed between black arrows. B- Slide stained with Picrosirius Red showing adhesion between the liver and the stomach, indicated between black arrows. C - Slide stained with Hematoxylin and Eosin showing adhesion between the liver, stomach and abdominal wall, shown between black arrows. D- Slide stained with Hematoxylin and Eosin showing adhesion between the liver, stomach and abdominal wall, pointed between arrows black, associated with liver abscess.

In the surgery group, the evaluation of slides stained with HE (Figures 6A, 6C and 6D) showed adhesions with large amounts of inflammatory cells, the presence of newly formed vessels, and some multinucleated giant cells, and the stained slides with RP under polarized light showed the presence of collagen fibers between the liver and the stomach, which appeared dark pink in color without the use of this type of light (Figure 6B). Figures 6C and 6D depict the microscopical evaluation of adhesions encompassing the abdominal wall, and Figure 6D shows a liver abscess in addition to the adhesion.

Two rats in the surgery group had liver abscesses diagnosed microscopically. No culture examination was performed on the abscess material and, therefore, the etiology of the abscesses is unknown. Both animals had a single abscess and there were no signs of suture dehiscence. These animals showed no signs of illness or pain.

In the sham group, when evaluating the slides stained with HE, the adhesions showed little infiltration of inflammatory cells and little or no neovascularization. When evaluating the slides stained with PR under polarized light, few collagen fibers were noted.

## DISCUSSION

Adhesions are abnormal bands of connective tissue with the presence of collagen, caused by impaired degradation of deposited fibrin, forming non-anatomical connections between organs or tissues^1^ ^,^ ^6^ ^,^ ^9^. There are countless factors that can induce the formation of peritoneal adhesions, such as infectious and inflammatory processes, pre-existing diseases, surgical trauma, proliferative diseases, radiation, presence of a foreign body, bleeding or clots, and the surgical technique^1^ ^-^ ^3^ ^,^ ^6^ ^,^ ^7^ ^,^ ^10^.

The formation of adhesions results from an imbalance between fibrin deposition and its degradation in the peritoneal tissues, with a reduction in fibrinolytic activity being the main alteration that leads to the formation of peritoneal adhesions^3^ ^,^ ^8^ ^-^ ^10^ ^,^ ^12^ ^-^ ^14^ ^,^ ^17^. This remaining fibrin is the basis for cell proliferation, collagen deposition, and vascular and neuronal growth^12^ ^,^ ^17^. When peritoneal adhesions become well organized and vascularized, they are considered permanent^12^. Ischemia is another important factor in the formation of peritoneal adhesions by inducing fibrin deposition^1^ ^,^ ^3^ ^,^ ^6^ ^,^ ^9^ ,and like inflammation, it may suppress fibrinolytic activity by overexpression of plasminogen activator inhibitors 1 and 23.

Peritoneal adhesions are an important cause of postoperative morbidity, chronic pain, female infertility, postoperative abdominal complications, and death, with great socioeconomic impact^1^ ^-^ ^11^. The formation of adhesions is the most common postoperative complication after operations in the peritoneal cavity, being the main cause of reoperations in the first ten years after the initial surgery^3^.

In an attempt to reduce surgical trauma, there should be delicate handling of the organs during the surgical procedure, strictly necessary dissection, constant humidification of intra-abdominal organs, strict hemostasis, in addition to sutures with adequate tension and prevention of ischemia and of infections. These principles were introduced by William S. Halsted and are the basic principles of good surgical technique^3^ ^,^ ^6^ ^,^ ^18^ ^,^ ^19^.

The incidence of postoperative adhesions varies, being found in 67% to 95% of patients submitted to previous laparotomy^7^ ^,^ ^9^ ^,^ ^10^, and it can reach up to 45% when accounting only for laparoscopic procedures^9^. Adhesions can also be found in 10.4% to 28% of patients who have never undergone abdominal surgery^17^ ^,^ ^20^, that is, they are not associated with previous surgical trauma, and are found during the first abdominal surgery or necropsy.

Some experimental models in animals^1^ ^,^ ^2^ ^,^ ^4^ ^,^ ^5^ ^,^ ^10^ ^-^ ^15^ have been used to induce peritoneal adhesions, but most of these experiments create adhesions through surgical procedures that, in everyday surgical practice, would be avoided as much as possible, since they go directly against Halsted’s surgical principles and his guidelines for minimizing trauma.

To give the models used in the literature as examples, intentional trauma to the uterine horn should not be performed, as reported by Harris et al.^14^, as it causes unnecessary damage and possibly bleeding, nor trauma to the serosa of the intestinal loops^1^ ^,^ ^2^ ^,^ ^5^ ^,^ ^14^. To avoid the formation of adhesions, the most delicate handling possible of the structures is indicated, in addition to optimizing the use of suture threads and abstaining from the use of powdered gloves, among other guidelines. Therefore, when animal models of induction of peritoneal adhesions are based on procedures that one tries as hard as possible to avoid, little correlation will be obtained with the main causes of postoperative adhesions encountered in routine surgical practice.

The model adopted by Thomas & Rhoads^11^ opts for the resection of a rectangular section of the serous layer of the cecum, with and without performing oversuture using silk thread, but this model was not able to cause adhesions in all animals, reaching the formation of adhesions in 74% and 28.5% of the animals, respectively. Silk thread was chosen, even though the authors suspected that this type of thread could induce the formation of peritoneal adhesions^11^. In that publication, extensive dissection was performed with the sole intention of causing damage intense enough to form adhesion, contrary to the surgical principles of minimizing damage and failing to form adhesions in all animals.

When comparing the methods to induce peritoneal adhesions, Nair et al.^12^ quote Thomas & Rhoads^11^ when saying that surgical resection of part of the intestinal serosa could not provide good results regarding the formation of adhesions, preferring the application of absolute alcohol in the serosa of the cecum, as the results were “uniformly satisfactory”, “quite constant”, and with “little chance of divergence” when they chose to use alcohol. This would never be adopted in medical practice, first because it does not have clinical applicability, and second due to the certainty of damage to the unicellular layer of the peritoneal mesothelium. Despite the toxic effects of alcohol, the results were not constant, as in some animals there was no formation of peritoneal adhesions, evidencing the lack of consistency of results^12^. These two authors were some of the pioneers in defining animal models for the induction of peritoneal adhesions and frequently have their models replicated or adapted to be used in research with peritoneal adhesions.

Cecal abrasion as a model for inducing peritoneal adhesions is probably the most frequently used method^1^ ^,^ ^2^ ^,^ ^5^ ^,^ ^14^. To increase the chance of creating adhesions, some authors associate parietal peritoneum abrasion above the cecum^4^ ^,^ ^10^ or resection of the peritoneum and muscle layer over the cecum, with^14^ or without fixation of the cecum close to the area of parietal peritoneum resection^5^. Cecal abrasion, until the removal of its serosa, in addition to not being able to cause adhesions in all animals, inflicts damage with the sole purpose of generate adhesions and increases the risk of fistulas, in addition to never being done in humans and therefore not being one of the most common causes of adhesion formation in surgical practice.

In addition to abrasion, cecal incision has been described, which has been associated with two deaths related to suture dehiscence^2^, isolated peritoneal abrasion^1^ ^,^ ^4^ ^,^ ^13^, isolated peritoneal excision^1^ ^,^ ^13^ ^,^ ^14^, or associated with suture of the peritoneal defect^4^, the peritoneal button^4^ ^,^ ^13^, peritoneum electrocautery with or without oversuture of the cauterized peritoneum^1^ ^,^ ^4^, uterine horn trauma^14^, peritonitis^14^, cecal abrasion with methanol^14^, and gastroenteroanastomosis^15^.

**Figure 7:**
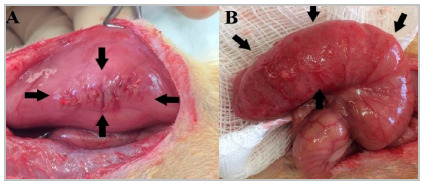
Pre-study rat, submitted to peritoneal electrocoagulation with suture and cecal abrasion. The black arrows point to the areas with damage. A- Electrocoagulation with suture. B- Cecal abrasion.

Before creating this animal model, we tested cecal abrasion associated with electrocautery of the contralateral parietal peritoneum, followed by oversuture of the cauterized area with 4.0 polyglactin thread. At necropsy, we oberved no peritoneal adhesions, although it was possible to notice macroscopic changes resulting from the procedures performed (Figure 7). We then tested gastroenterostomy^15^, but this model proved to be technically challenging as all the animals died between the second and fifth postoperative day.

Most of the models described in the specific literature exert their effect in a similar way, exponentially increasing the damage, so that they are able to form adhesions, regardless of the real reason for studying them, which is the elucidation of their pathophysiology or the search for ways of prevention. Exacerbating damage creates adhesions, but not how most adhesions are formed, and therefore, as the cause of the adhesion is distinct, its prevention may be as well. Thus, it is essential to use a model that induces postoperative peritoneal adhesions in all operated animals, and that renders adhesions with a consistent appearance for future comparison.

Then so a new model was developed for the induction of peritoneal adhesions by performing an operation on the stomach, which was submitted to an incision (gastrotomy) in its anterior wall, followed by immediate closure (gastrorrhaphy), mimicking a surgical procedure in the supramesocolic region and similar to how they are routinely performed in humans, that is, avoiding unnecessary or exaggerated damage. The results showed the formation of peritoneal adhesions in all animals in the surgery group, with no deaths.

For the analysis of adhesions, we opted for the Nair and Evans scales, which assess macroscopic aspects of adhesions by assigning scores, remembering that rupture by traction of adhesions was not attempted in our study, aiming to maintain the integrity of the adhesion for subsequent histopathological assessment, with evaluation and classification being performed only macroscopically. The microscopic results confirmed that the adhesions analyzed were completely formed, ruling out the possibility that they were non-degraded fibrin bands.

We believe that in the procedure described in our animal model, the constant contact of the liver with the stomach was one of the determining factors for the creation of stable peritoneal adhesions between these structures. On the anterior surface of the stomach, submitted to gastrotomy and subsequent gastric suture, there was the formation of a physiological inflammatory reaction, induced both by the injury inflicted on the gastric wall and by the body’s need to promote regeneration and healing of injured areas. Although the liver did not suffer direct trauma, the inflammatory reaction with its entire process of cell migration, fibrin deposition, and impaired fibrinolysis created collagen bridges between the two organs that were subsequently organized in the form of adhesions.

The most replicated animal models, in addition to being unable to form adhesions in all animals, when they do, do not show the same pattern, which prevents comparison since there is no predictability of what will be found. Moreover, they only seek the formation of adhesions and not that adhesions are formed in the same way as the ones formed in the surgical practice. The procedures used in these frequently replicated models would be considered medical malpractice in humans. The model described in this article replicates an operation that exists and is performed all over the world and, for the gastrotomy and gastrorrhaphy to be performed, the utmost care is indicated so that only the gastric incision is the factor causing injury and, consequently, the adhesions.

One must also consider why animals are able to develop adhesions when peritoneal structures are injured. The complete inhibition of the formation of peritoneal adhesions is antiphysiological and potentially dangerous, since they may have allowed some animals to survive traumatisms or inflammatory processes, preventing the dissemination of the offending agent through the peritoneal cavity and allowing the evolution of the species. The search for substances that modulate, and do not nullify, the formation of peritoneal adhesions may be the key to reducing the morbidity and mortality caused by this disease. It is thus extremely important to have a suitable animal model to induce peritoneal adhesions, which can lead to the formation of adhesions whenever adopted, follows the principles of not causing exaggerated and unnecessary damage, and is safe for the animals.

## CONCLUSION

The model of gastrotomy followed by gastrorrhaphy proposed in this work induced the formation of extensive, dense, and stable peritoneal adhesions in all operated animals, without the use of techniques that need to cause excessive damage to achieve their intent. The great differentiating factor of this model from all others researched in the literature is the constant expected result (adherence of the stomach and omentum to the posterior aspect of the liver), varying only in volume. Therefore, the new model presented proved to be safe and efficient to induce and study peritoneal adhesions.
